# The Dilemma of Renal Involvement in COVID-19: A Systematic Review

**DOI:** 10.7759/cureus.8632

**Published:** 2020-06-15

**Authors:** Hamza Bajwa, Yumna Riaz, Muhammad Ammar, Soban Farooq, Amman Yousaf

**Affiliations:** 1 Internal Medicine, King Edward Medical University, Mayo Hospital, Lahore, PAK; 2 Internal Medicine, Allama Iqbal Medical College, Lahore, PAK; 3 Radiology, Hamad General Hospital, Doha, QAT; 4 Radiology, Services Institute of Medical Sciences, Lahore, PAK

**Keywords:** acute kidney insufficiency, coronavirus, cytokine release syndrome, renal replacement therapy, dialysis, chronic kidney disease

## Abstract

Severe acute respiratory syndrome coronavirus 2 (SARS-CoV-2), now known as coronavirus disease 2019 (COVID-19), has posed a serious threat to global health since December 2019. It has spread worldwide and is consuming healthcare resources rapidly. Published literature suggests that people with advanced age and comorbidities are affected more severely. It is crucial to uncover the underlying pathogenesis of acute kidney insufficiency in COVID-19 patients to understand better the reasoning behind the grave outcomes in these patients. In this review, we have included articles stating the prevalence and specific mortality rates of COVID-19 patients with acute kidney insufficiency. Our study included 1098 COVID-19 positive patients, of whom 66 (6%) developed acute kidney insufficiency and 62 patients died, showing a mortality rate of 94%. Patients with acute kidney insufficiency showed a more severe disease course, and these patients ended up more in intensive care units. Particular attention should be paid to those with already established kidney disease, such as chronic kidney disease, or renal transplant recipients, as these patients are already on immunosuppressive therapy. Our review focuses on the prevalence of acute kidney insufficiency in COVID-19 patients and mortality rates in this subset of patients.

## Introduction and background

The turn of the decade brought forth a new challenge for the entire world. This time the enemy was elusive, a minute particle made up of protein and nucleic acid. In December 2019, coronavirus hit the world with its third major epidemic [1]. Starting from Wuhan, in the Hubei province of China, it spread to other countries and became a global problem within months. On February 11, 2020, the World Health Organization (WHO) named this illness as coronavirus disease 2019 (COVID-19) and later declared it a pandemic on March 11, 2020 [2]. The western world has been most affected by this illness, with peaks noted in European countries and the United States (US). Until May 8, 2020, 1,256,994 cases have been reported in the US, and 3,835,107 cases globally, with 1,283,029 patients recovered and 268,340 reported deaths worldwide [3].

There is a wide range of clinical presentations of COVID-19-infected patients, varying from asymptomatic recovery to critical illness and death. Classically, patients with COVID-19 present with cough, fever, dyspnea, fatigue, and respiratory failure along with multiorgan damage in severe cases [4-6]. The disease is highly contagious and spreads in cluster outbreaks. Person-to-person contact via respiratory droplets is the primary mode of transmission. The fecal-oral route might be possible, while aerosol, tear, semen, and mother-to-fetus transmission are yet to be confirmed [7].

There are several studies in the literature illustrating the possible mechanism of dissemination and multiorgan involvement in COVID-19 patients. So far, diffuse alveolar damage and acute respiratory failure are the main features of severe COVID-19, but the data on the kidney's involvement is limited. Initial reports from Wuhan suggested that the prevalence of acute kidney injuries (AKI) in COVID-19 patients was quite low, ranging from 3-9%; however, the subsequent analyses showed a relatively high AKI burden of 15% [8]. Another Chinese cohort study of 1099 patients with COVID-19 revealed that only 0.5% developed AKI [9]. While some studies have shown this association, data on the patients' mortality is limited. Chen et al. reported an incidence rate of 6.7 % and the mortality rate of 91.7 % of AKI in severe acute respiratory syndrome [2]. Nevertheless, a study on the association of renal injuries with COVID-19 is warranted. Our focus in this review is to analyze the published data on kidney injuries in COVID-19 and mortality rates in these patients.

## Review

Materials and Methods

**Figure 1:**
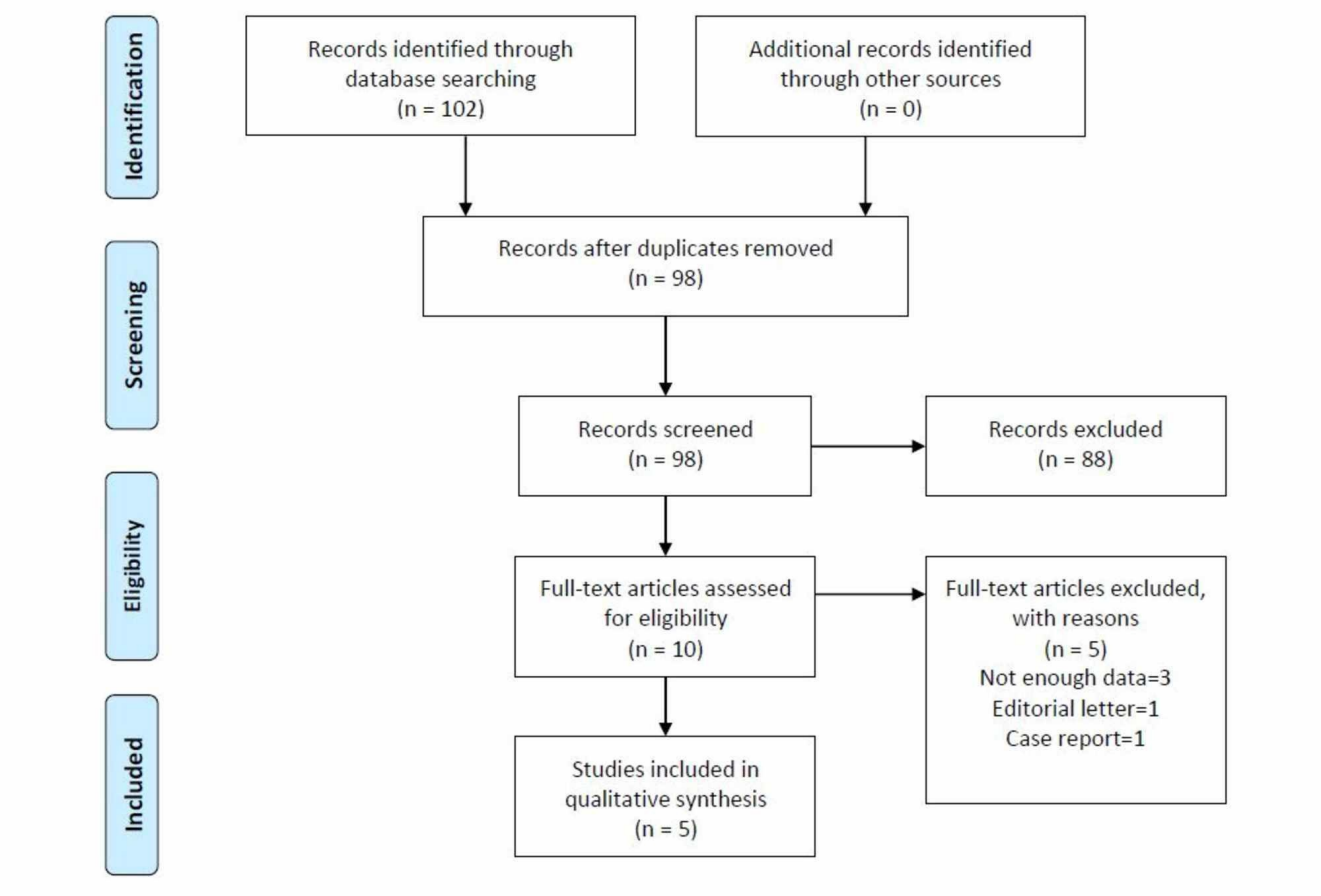
PRISMA flow diagram The PRISMA diagram details our data identification, screening, eligibility testing, and inclusion according to PRISMA guidelines. PRISMA: Preferred reporting items for systematic reviews and meta-analyses

A systematic search was done on PubMed, Cochrane Library, ClinicalTrials.gov, and Google Scholar following preferred reporting items for systematic reviews and meta-analyses (PRISMA) guidelines [10] (Figure 1)

The search was performed using MeSH terms, “acute kidney injury” AND “coronavirus”. Our search was not limited to any geographical area, and all relevant published articles in English or English translation from December 2019 to April 13, 2020, were included in our study.

Results

We identified 102 articles through systematic searches. Out of these, five articles were analyzed and included in our review. These five articles had a pool of 1098 COVID-19 positive patients. Twenty-three patients already had chronic kidney disease (CKD), and seven patients were kidney transplant recipients (TRx). Out of 1098 total patients, 66 (6%) developed AKI after the acquisition of COVID-19. However, 62 out of these 66 patients died, showing a high mortality rate of 94%. Out of 1032 patients who did not develop AKI, 172 patients died, and 860 recovered (mortality rate= 17%). The results of individual studies are explained (Table 1).

**Table 1:** Baseline characteristics and comparison of results Values are given in numbers (percentages). M, Male; F, Female; Yr, Year; CKD, Chronic kidney disease; TRx, Transplant recipient; AKI, Acute kidney injury; COVID-19, Coronavirus disease 2019; BUN, Blood urea nitrogen; Cr, Creatinine; eGFR, Estimated glomerular filtration rate; IVIG, Intravenous immunoglobulin. [2], [4], [11-13]

Author /Year	Cheng Y, et al. (2020)	Chen T, et al. (2020)	Gandolfini I, et al. (2020)	Wang L, et al. (2020)	Zhang H, et al. (2020)	Total
Study location	China	China	Italy	China	China	
Number of patients	701	274	2	116	5	1098
M	367 (52%)	171 (62%)	1 (50%)	67 (58%)	4 (80%)	610 (55%)
F	334 (48%)	103 (38%)	1 (50%)	49 (42%)	1 (20%)	488 (45%)
Median age (Yr)	63	62	64	54	45	
Patients with known CKD or TRx	14 (2%)	4 (1%)	2 (100%)	5 (4%)	5 (100%)	30 (3%)
Patients with AKI after COVID-19 infection	36 (5%)	29 (10%)	1 (50%)	0 (0%)	0 (0%)	66 (6%)
On admission: BUN/Cr (mg/dL) (mean)	-	13.72 / 0.86	- / 2.3	With CKD: 89.83±24.03 / 10.62±1.34 Without CKD: 14.65±4.82 / 0.88±0.29	33.27±20.08 / 2.10±1.44	
Change in BUN/Cr (mg/dL) (mean)	-	23.52 / 0.99	- / 2.8	With CKD: 89.44±25.71 / 10.34±1.85 Without CKD: 14.53±5.80 / 0.82±0.28	-	
Change in eGFR (mL/min) (mean)	-	-	-	With CKD: 14.43±7.34 to 22.86±9.37 Without CKD: 129.81±10.33 to 127.96±9.65	-	
Mortality with AKI	34 (94%)	28 (96%)	0 (0%)	0 (0%)	0 (0%)	62 (94%)
Mortality without AKI	79 (12%)	85 (35%)	1 (100%)	7 (6%)	0 (0%)	172 (17%)
Major treatment provided (including mechanical ventilation)	Arbidol, interferon, lopinavir and ritonavir, glucocorticoids, antibiotics	Antivirals, glucocorticoids, IVIGs, antibiotics, interferon	Hydroxychloroquine, lopinavir + ritonavir, or darunavir + cobicistat, colchicine	-	Antiviral (oseltamivir or arbidol), antibacterial therapy (cefixime), IVIGs, triple immunosuppression with glucocorticoids, mycophenolate mofetil and calcineurin inhibitors	

Cheng et al. enrolled 701 COVID-19 positive patients admitted in Tongji Hospital, Wuhan, to study the prevalence of AKI in COVID-19 patients and to determine the association between abnormal kidney function markers and mortality in COVID-19 patients. Statistical analysis of the data showed an incidence of AKI in 5.1% of all enrolled patients and a much higher incidence (11.9%) of AKI in patients who already had elevated baseline serum creatinine levels at the time of admission. The analysis also revealed a higher incidence of in-hospital mortality (33.7%) in COVID-19 patients with raised baseline serum creatinine levels as compared to mortality (13.2%) in those with normal baseline serum creatinine levels at the time of hospitalization. The review of medication data showed a higher trend of treatment with antiviral drugs (*p *= 0.041) and glucocorticoids (*p *= 0.006) in COVID-19 patients who developed AKI as compared to COVID-19 patients without AKI. Moreover, COVID-19 patients who developed AKI showed a high treatment trend with diuretic drugs (p<0.001) during hospitalization compared to those without AKI [2].

Chen et al. studied the comprehensive clinical characteristics of patients with COVID-19 at Tongji Hospital, Wuhan, China. Out of 799 COVID-19 patients, the data was available for 274 patients. A total of 113 patients died (41%) due to COVID-19, and 161 patients (59%) fully recovered. Out of 274 patients, four patients had CKD, and 29 patients (10%) developed AKI after the COVID-19 infection. Deceased patients had more increase in blood urea nitrogen (BUN) concentration (median = 23.52 mg/dL) than the recovered patients (median = 13.72 mg/dL). Creatinine concentration was also reported higher in deceased patients (median = 0.99 mg/dL) than the recovered patients (median = 0.86 mg/dL). Out of 274 patients, 100 patients (37%) developed proteinuria, of which 42 were deceased, and 58 patients fully recovered. Urinary occult blood was positive in 84 cases (30%), out of which 44 were deceased, and 40 recovered. Hyperkalaemia was reported in 42 (37%) of 113 diseased patients. Patients were treated with monotherapy or combination therapy with antiviral agents (oseltamivir, arbidol, or lopinavir/ritonavir). According to the study, recovered patients received more combination therapy (91%) than deceased patients (79%). However, glucocorticoid therapy was given more to deceased patients (88%) than the recovered patients (73%) [11].

Wang et al. recruited 116 hospitalized COVID-19 patients with a median age of 54 years. Associated comorbidities included hypertension, diabetes, malignant tumors, cerebral infarction, and CKD. Statistical analysis of results showed a mild increase in BUN or creatinine (< 0.3 mg/dL) in 12 patients, and eight patients showed +1 albuminuria, but none of them met the criteria of AKI. Moreover, five patients who already had CKD did not show any sign of CKD exacerbation. Change in BUN in patients without CKD in the first four weeks was 14.65±4.82, 14.66±4.64, 13.79±3.37, 14.53±5.80 (mg/dL) respectively. Similarly, change in creatinine was 0.88±0.29, 0.89±0.31, 0.86±0.28, 0.82±0.28 (mg/dL) respectively. The change in BUN in patients with CKD in four weeks was 89.83±24.03, 88.92±24.44, 89.78±25.54, 89.84±25.71 (mg/dL) respectively. Change in creatinine was 10.62±1.34, 11.72±1.44, 9.24±1.16, 10.34±1.85 (mg/dL) respectively. Change in estimated GFR (mL/min) from admission to four weeks was 14.43±7.34 to 22.86±9.37 with CKD and 129.81±10.33 to 127.96±9.65 without CKD. The p-value for all these results was >0.05. All patients recovered gradually without receiving special treatment for kidneys. The temporary renal impairment was supposed to be from hypoxemia caused by acute respiratory distress syndrome (ARDS) or pneumonia [13].

Gandolfini et al. studied the disease course of COVID-19 on two renal transplant patients. Both patients had received deceased-donor grafts. The first patient was a 75-year-old male who received renal transplant ten years back and was on tacrolimus, steroid, and mycophenolate. His graft function was stable at the time of admission, with a baseline creatinine of 2.1 mg/dL. Mycophenolate and tacrolimus were withheld at that time, and he was started on hydroxychloroquine with combination therapy of lopinavir+ritonavir or darunavir+cobicistat. His condition worsened suddenly over 24-48 hours, and he expired five days after the admission. His creatinine concentration remained stable, ranging between 2.1-2.2mg/dL. The second patient was a 52-year-old female; she received her renal transplant eight months back from a deceased donor and was stable on steroid, mycophenolate, and tacrolimus. At the time of admission, she had already developed AKI, and her creatinine concentration ranged between 2.4 to 3.4 mg/dL as compared to her baseline creatinine concentration of 1.3mg/dL. Mycophenolate and tacrolimus were suspended, and she was started on hydroxychloroquine and a combination of lopinavir+ritonavir or darunavir+cobicistat. On day six of admission, her condition worsened, and she was given colchicine to reduce systemic inflammation. The patient remained stable after that episode, and her serum creatinine concentration returned to her baseline level of 1.4mg/dL [12].

Zhang et al. studied the effect of COVID-19 on five kidney transplant recipients (four males, one female). The median age of patients was 45 (range: 34-56) years. Associated comorbidities included hypertension, diabetes mellitus, and bladder cancer. The study showed that baseline BUN and creatinine were further increased after the onset of COVID-19 symptoms. The mean BUN and creatinine levels at the time of admission were 33.27±20.08 mg/dL and 2.10±1.44 mg/dL, respectively. Patients were managed symptomatically, and immunosuppressive medications dosage were decreased. All five patients recovered successfully, and no incident of AKI directly caused by COVID-19 was reported. However, one patient developed acute kidney rejection due to lowered immunosuppressive doses, but that resolved after switching back to the maintenance dosage [4].

Discussion

Our review comprehensively described the prevalence of acute kidney injury (AKI) in COVID-19 patients. The studies included were two prospective cohorts, one retrospective, and two case-series. The incidence of AKI was high in patients with elevated baseline serum creatinine (Cr). AKI of higher than stage II and Cr > 1.5 mg/dL were associated with in-hospital death of COVID-19 patients [2]. The admission to intensive care units (ICU) and mechanical ventilation requirements were also higher in patients with raised baseline Cr regardless of COVID-19 severity and general physical condition on admission. The AKI patients have a higher mortality rate than patients with other comorbidities. It is observed to be more than the mortality rates of patients with cardiovascular pathologies (58 %) [11]. Therefore, kidney functions should be monitored in patients with even mild respiratory symptoms, and particular attention must be given to those with altered kidney functions. 

Various mechanisms have been proposed to explain how COVID-19 infection causes acute kidney injury (AKI). Cytokine release syndrome (CSR) offers a reasonable explanation for this association. CRS can lead to AKI by causing intrarenal inflammation, increased vascular permeability, volume depletion, and cardiomyopathy, leading to cardiorenal syndrome type 1. The syndrome includes systemic endothelial injury, which manifests clinically as pleural effusions, edema, intra-abdominal hypertension, third-space fluid loss, intravascular fluid depletion, and hypotension. Interleukin 6 (IL-6) is a pro-inflammatory cytokine and has a major role in CRS. The level of IL-6 is markedly increased in COVID-19 patients with acute respiratory distress syndrome (ARDS). The anti-IL-6 monoclonal antibody tocilizumab is widely used to treat CRS in many other medical conditions and is now being used empirically in severe COVID-19 cases [9]. The support for critically ill COVID-19 patients with extracorporeal membrane oxygenation (ECMO), invasive mechanical ventilation, and continuous kidney replacement therapy (CKRT) also contributes to the generation of cytokine leading to increased chances of AKI in critically ill patients. ARDS also results in additional damage to tubular cells in renal medulla by hypoxic injury [9, 14-15].

The usual practice of fluid expansion in patients with shock may have a detrimental effect on kidneys, as it can worsen renal vein congestion, leading to renal compartment syndrome. A similar picture might be expected in COVID-19; however, recent studies do not have this association. Hyperkalemia, rhabdomyolysis, and metabolic acidosis can also occur in COVID-19 patients and almost always are associated with hemodynamic instability. A recent study has encouraged the use of continuous renal replacement therapy (CRRT) in these patients, with or without medium cut-off or high cut-off membranes [9]. In extended ICU stays, superimposed infections can cause septic shock. In a Chinese study of 1099 patients, sepsis was present in 11 of 173 patients (6.4%) with severe COVID-19 [16]. We can assume that septic AKI may occur in such patients and act synergistically with other mechanisms of kidney injuries. In patients with suspected or confirmed gram-negative bacterial infections and an endotoxin activity assay result of 0.6-0.9, the use of hemoperfusion with a cartridge containing polystyrene fibers functionalized with polymyxin-B provides effective endotoxin adsorption. The functionalized surface has sites that bind to endotoxin, reducing its plasma concentration [9].

Patients on hemodialysis seem to represent another target population for severe COVID-19 disease. However, data does not suggest a variation from the general population until regular maintenance hemodialysis is provided. A study on this association showed 5 out of 201 maintenance hemodialysis patients contracted COVID-19, and none of them developed serious complications or died [17]. Renal transplant recipients (TRx) are another group of susceptible populations because they are on chronic immunosuppression, making them more susceptible to pathogens and infections. These patients have a high trend of ending up in intensive care units (ICU). An important consideration is how many patients will require renal replacement therapy or dialysis as the need for dialysis usually arises around the second week of infection, and about 5% of ICU patients require dialysis [8]. However, in our review, TRx did not show complications related to COVID-19, and contrary to this, all five patients in the study conducted by Zhang et al. survived, and none of them developed the severe disease [4]. There is no need to lower immunosuppressive dose in TRx with mild COVID-19 symptoms, while drug adjustment is needed in those who develop acute respiratory distress syndrome or pneumonia [8, 12]. Early detection of renal derangements and treatment may improve the prognosis of COVID-19 patients. Therefore, to improve early detection of renal injuries, more frequent serum creatinine measurements should be performed.

Our review was limited regarding the number of articles. As it is still a new disease, data on the association of AKI with COVID-19 is limited. We could only include three articles directly revealing this association, the population in the other two articles was renal transplant recipient. We suggest more studies should be conducted on the prevalence and mortality of AKI patients diagnosed with COVID-19.

## Conclusions

Our study shows that the incidence rate of AKI in COVID-19 patients is around 6%, which is not alarming if we compare the incidence rate of other organ injuries, such as lungs and heart, in COVID-19. However, the alarming fact is the high mortality rate of 94% in these patients. These results demand that patients who are prone to developing AKI should be given special attention, and kidney functions should be monitored frequently. In kidney transplant recipients and those with chronic kidney disease, the mortality rate is also low until AKI develops. These results are from a limited data pool, and hence, we suggest conducting further studies involving a larger patient population to further evaluate this association.
